# Protective effects of α-terpineol and *Bacillus coagulans* on intestinal function in weaned piglets infected with a recombinant *Escherichia coli* expressing heat-stable enterotoxin STa

**DOI:** 10.3389/fvets.2023.1118957

**Published:** 2023-02-10

**Authors:** Tao Wu, Qian Zhang, Haiwang Xu, Peng Li, Di Zhao, Lei Wang, Dan Yi, Yongqing Hou

**Affiliations:** Hubei Key Laboratory of Animal Nutrition and Feed Science, Engineering Research Centre of Feed Protein Resources on Agricultural By-Products, Ministry of Education, Wuhan Polytechnic University, Wuhan, China

**Keywords:** *B. coagulans*, α-terpineol, *Enterotoxigenic Escherichia coli*, intestinal function, antibiotic alternatives, weaned piglets

## Abstract

This study was to investigate the impact of α-terpineol (α-TPN) and *Bacillus coagulans* (*B. coagulans*) on weaned piglets infected with *Enterotoxigenic Escherichia coli* (ETEC). Thirty-two weaned piglets were assigned into four treatments: Control group (basal diet), STa group (basal diet + 1 × 10^10^ CFU ETEC), TPN+STa group (basal diet + 0.01% α-TPN + ETEC) and BC+STa group (basal diet + 2 × 10^6^ CFU *B. coagulans* + ETEC). Result showed that both α-TPN and *B. coagulans* could alleviate diarrhea (decreased diarrhea rate), intestinal injury (improved intestinal morphology, decreased blood I-FABP concentration, increased protein expression level of Occludin), oxidative stress (increased GSH-Px activity and decreased MDA content) and inflammation (altered concentration of TNF-α, IL-1β in blood) induced by ETEC infection. Mechanism investigation further demonstrated that the beneficial effects of α-TPN and *B. coagulans* supplementation upon ETEC infection may be achieved by decreasing the protein expression levels of caspase-3, AQP4 and p-NF-κB and decreasing the gene expression levels of *INSR* and *PCK1*. Besides, α-TPN supplementation could specifically decreased expression level of gene *b*^0,+^*AT*, and *B. coagulans* supplementation could specifically decreased expression level of gene *AQP10* and protein HSP70 in ETEC-infected weaned piglets. These results suggested that α-TPN and *B. coagulans* can be used as antibiotic alternatives against ETEC infection in weaned piglets.

## 1. Introduction

*Enterotoxigenic Escherichia coli* (ETEC) strains are the most common bacterial pathogens that cause diarrhea, which is one of the most important diseases in young farm animals, also the second leading cause of death in children under 5 years old (1, 2). ETEC-induced post-weaning diarrhea in piglets mainly occurs within the 1st week after weaning (3). ETEC infection could result in great economic losses to the swine industry due to reduced growth performance as well as increased morbidity and mortality of piglets. Over two thirds of diarrhea in piglets caused by ETEC are resulted from STa (heat-stable toxin A)-producing ETEC strains, thereby the most heated studies of ETEC frequently focus on STa (4). In order to study the pathology and toxicology of STa, a recombinant *E. coli* expressing STa, named LMG194-STa, was constructed by using *E. coli* strain LMG194 and the plasmid pBAD202/Dtopo in our previous work. *E. coli* LMG194-STa has been demonstrated to induce diarrhea and intestinal injury in piglets (5, 6).

Since the use of antibiotic in livestock has been reducing in China, many studies on piglet diarrhea have devoted to exploring the possible alternatives to antibiotics against ETEC infection (7). For which, essential oils and probiotics have been considered as ideal alternatives to antibiotics, due to their natural activities against pathogenic bacteria. Plant essential oils contain a wide variety of secondary metabolites that can inhibit or slow the growth of bacteria, yeasts, and molds (8). α-Terpineol (α-TPN) is a volatile monoterpene alcohol, a major component of the essential oils from various plant species (9). Research has demonstrated that it has insecticidal, antimicrobial, antispasmodic, anticonvulsant, antinociceptive and immunostimulant properties (10). It is present in the essential oil of several medicinal plants, such as *Punica granatum* L. (Pomegranate), *Rosmarinus officinalis* L. (Rosemary), *Psidium guajava* L. (Guava), which is popularly used for treating diarrhea. The antidiarrheal activity of α-TPN has been evaluated in mice (11). This evidence suggested that α-TPN could be used to reduce diarrhea and resist ETEC infection and STa toxin.

*Bacillus coagulans* (*B. coagulans*) is a lactic acid producing bacterial species, which is catalase positive, spore forming, motile, and a facultative anaerobe (12). Because of the strong resistance, resurrection, and stability, spores of *B. coagulans* could be activated in the acidic environment of the stomach, then germinate and proliferate in small intestines (13). Such spores could adapt to the gastrointestinal environment and reach the gut smoothly, and thereby play the beneficial impacts in the intestines (13). Due to these characteristics, *B. coagulans* has been frequently used in animal nutrition, particularly as a probiotic in poultry, cattle and shrimp (14). Our previous works have also demonstrated that supplementation of *B. coagulans* modulated metabolism and microbiome, alleviate intestinal integrity, oxidative stress and diarrhea (15), which may be also a good option for the treatment of ETEC infection.

Based on our previous studies on the recombinant *E. coli* LMG194-STa, the present study was conducted to investigate the protective effects of α-TPN and *B. coagulans* on intestinal function in weaned piglets infected with LMG194-STa, thereby further exploring the possible mechanisms. Findings of this study can contribute to the development of alternatives to antibiotics, and provide a novel strategy against ETEC infection.

## 2. Material and methods

### 2.1. Animals and design

The animal use protocol for the present study was approved by the Animal Care and Use Committee at Wuhan Polytechnic University (0328-2017-0117). Thirty-two crossbred healthy piglets (Duroc × Landrace × Yorkshire) were weaned at 21 days of age. Each piglet was individually housed in a 1.20 × 1.10 m^2^ steel metabolic cage with eight replicates per treatment. After weaning, piglets had a 3-day adaptation. During this period, piglets had free access to the basal diet for adapting to solid food. Then piglets were assigned randomly on the basis of body weight (5.62 ± 0.66 kg) and litter origin to 4 groups: 1) control group-piglets were fed the basal diet; 2) STa group-piglets were fed the basal diet and challenged with 1 × 10^10^ *E. coli* LMG194-STa; 3) TPN+STa group-piglets were fed the basal diet supplemented with 0.01% α-TPN, and challenged with 1 × 10^10^ CFU *E. coli* LMG194-STa and; 4) BC+STa group-piglets were fed the basal diet supplemented with 2 × 10^6^ CFU *B. coagulans*, and challenged with 1 × 10^10^ CFU *E. coli* LMG194-STa. The composition and nutrient levels of the basal diet are shown in Table 1. From day 1 to day 10 of the trial, piglets were fed the basal diet with or without α-TPN or *B. coagulans*. All diets were isocaloric. On day 11 and day 12, each piglet was orally inoculated with 1 × 10^10^ CFU of *E. coli* LMG194-STa at both 8:00 and 20:00. The dose of ETEC and *B. coagulans* were determined based on our previous studies (15, 16). The dose of α-TPN was chosen according to the result of a preliminary experiment. The recombinant *E. coli* LMG194-STa were constructed by our previous work, and cultured in Lysogeny broth liquid medium (5, 6). *B. coagulans* were produced by fermenting; the conditions were 200 r/min of stirring speed and 350 L/h of throughput. The products of fermentation were filtered by organic ceramic membrane filtration equipment, then mixed with mineral adsorbent by 1:1. Final products were a dry powder that were counted by the plate counting method to determine the dosage and then were mixed in the diet. On the 15th day of the trial, blood samples were collected from the anterior vena cava and transferred into a tube with heparin sodium for anticoagulation. Plasma and serum were separated by centrifugation at 3,500 rpm for 15 min and stored at −20°C. All piglets were then slaughtered under anesthesia. Intestine, and intestinal content were collected and stored at −80°C until assay.

**Table 1:** Composition and nutrient levels of the basal diet.

**Items**	**Content (%)**	**Items**	**Content (%)**
Ingredients		Nutrient level^b^	
Corn	38.40	Metabolizable energy(MJ/kg)	14.27
Soybean meal	16.00	CP	18.54
Flour	12.00	Lys	1.50
Whey powder (low Protein)	8.00	Met	0.42
Soybean protein concentrate	5.00	Met+Cys	0.71
Wheat middling	5.00	Thr	0.93
Fish meal	4.50	Trp	0.23
Glucose	3.00	Ca	0.75
CaHPO_4_	1.33	AP	0.49
Limestone	0.37	TP	0.68
NaCl	0.25	Na	0.31
Plant oil	3.95	CF	6.26
Premix^a^	1.00	NaCl	0.61
Lys	0.64		
Met	0.13		
Thr	0.21		
Choline	0.12		
Mildew preventive	0.10		

a Premix provided the following amounts of vitamins and trace minerals per kilogram of diet: Fe 100 mg, Cu 150 mg, Mn 40 mg, Zn 100 mg, I 0.5 mg, Se 0.3 mg, VA 10 800 IU, VD_3_,4,000 IU, VE 40 IU, VK_3_ 4 mg, VB_1_ 6 mg, VB_2_ 12 mg, VB_6_ 6 mg, VB_12_ 0.05 mg, Biotin 0.2 mg, Folic acid 2 mg, Niacin 50 mg, D-Calcium pantothenate 25 mg.

b Nutrient levels were analyzed values except for metabolizable energy.

### 2.2. Plasma parameters analysis

The activity of superoxidase dismutase (SOD) and glutathione peroxidase (GSH-Px), and the content of malondialdehyde (MDA) in plasma were determined by spectrophotometry using commercially available kits (Jiancheng Bioengineering Institute, Nanjing, China). The concentration of tumor necrosis factor alpha (TNF-α), interleukin 1 beta (IL-1β), and intestinal fatty-acid binding protein (I-FABP) was determined by ELISA using Quantikine^®^ Assay Kits (Fisher Scientific Inc., Minneapolis, MN, USA). Assays were performed in triplicate.

### 2.3. Intestinal morphology

Intestinal tissue samples used for the morphometric study were dehydrated and embedded in paraffin, sectioned at a thickness of 4 mm, and stained with haematoxylin and eosin. Morphological measurements were carried out using a light microscope (Leica microsystems, Wetzlar, Germany) equipped with the Leica Application Suite image analysis software (Leica microsystems, Wetzlar, Germany). Intestinal villus height and width, as well as crypt depth, were measured to calculate both the villus crypt ratio and villous surface area.

### 2.4. Real-time PCR

The gene expression levels were quantitated by the method of real-time PCR. Firstly, RNA from the frozen samples were isolated using the Trizol Reagent protocol (Invitrogen, Carlsbad, CA). Then cDNA was synthesized using a PrimeScript^®^ RT reagent kit (Takara, Dalian, China) with gDNA Eraser. Finally, the real-time PCR was carried out with the SYBR^®^ Premix Ex Taq™ (Takara, Dalian, China) on 7,500 Fast Real-Time PCR System (Applied Biosystems, Foster City, CA, USA). Ribosomal protein L4 (RPL4) was used as the reference gene. Data was analyzed by using the 2^−ΔΔCt^ method as described previously (17). The primer sequences are shown in Table 2.

**Table 2:** Primer sequences used in the present study.

**Genes**	**Forward (5'-3')**	**Reverse (5'-3')**
Villin	AGAAGTGGACGGTGCCCAAC	TCTCGCCGATGAGGTAGGTG
MMP3	GATGTTGGTTACTTCAGCAC	ATCATTATGTCAGCCTCTCC
I-FABP	AGATAGACCGCAATGAGA	TCCTTCTTGTGTAATTATCATCAGT
*b^0,+^AT*	CGAGTACCCGTACCTGATGGA	TGCGTAGAAGGGCGAAGAA
AQP10	GGGCGTTATACTAGCCATCTAC	CCAACTGCACCAAGGAGTAA
KCNJ13	ATGGATGTGTCGCTGGTCTTT	CACAACTGCTTGCCTTTACGAG
INSR	GGGGCTAAAGAGGAACTATGAGG	AGAGGAAAGCGAAGACAGGAAA
LPL	AGCCTGAGTTGGACCCATGT	CTCTGTTTTCCCTTCCTCTCTCC
PCK1	CGGGATTTCGTGGAGA	CCTCTTGATGACACCCTCT
RPL4	GAGAAACCGTCGCCGAAT	GCCCACCAGGAGCAAGTT

### 2.5. Western blot

The expression levels of proteins were performed using western blot. Tissue extracts were prepared using RIPA buffer. After electrophoresis, proteins were transferred onto PVDF membranes. Then the membrane was blocked for 1 h at room temperature with 5% milk solution, and incubated with primary antibodies at 4°C overnight. The following antibodies were used in this study: NF-κB (6956S, Cell Signaling Technology), pNF-κB (3033, Cell Signaling Technology), Mx1 (ab79609, Abcam), IFN-α (ab230993, Abcam), HSP70 (ADI-SPA-810-F, Enzo Life Sciences), I-FABP (sc-16063, Santa Cruz Biotechnology), Caspase-3 (9661S, Cell Signaling Technology), Villin (sc-7672, Santa Cruz Biotechnology), AQP4 (ab46182, Abcam), AQP3 (ab125219, Abcam), Claudin (RF217968, Invitrogen), and Occludin (TC259714, Invitrogen), β-actin (PA1-46296, Invitrogen). Subsequently, appropriate HRP-conjugated secondary antibodies were used followed by incubation at room temperature for 1 h. Blots were visualized using a chemiluminescence kit (Amersham Biosciences, Uppsala, Sweden) and image forming system (Alpha Innotech, New York, NY, USA).

### 2.6. Statistical analysis

All data, expressed as means ± SD, were analyzed by one-way analysis of variance (ANOVA). The normality and constant variance for experimental data were tested by the Levene's test. Differences among treatment means were determined by Duncan's multiple range tests. All statistical analyses were performed using SPSS 24.0 software (Chicago, IL, USA). *P*-values < 0.05 were taken to indicate statistical significance.

## 3. Results

### 3.1. Growth performance

During the experimental period, the average daily gain (ADG), average daily feed intake (ADFI), and diarrhea rate (DR) was recorded and calculated (Table 3). There was no significant difference in ADG, ADFI and F/G among these treatments throughout the experiment. However, ETEC infection significantly increased diarrhea rate during day 11 to 15 of the trial, whereas the supplementation of both α-TPN and *B. coagulans* significantly alleviated diarrhea (*P* < 0.05).

**Table 3:** Growth performance of piglets.

**Item**	**Control**	**STa**	**TPN+STa**	**BC+STa**
**Day 1–10** ^c^				
ADG /g	135.5 ± 30.4	183.8 ± 26.3	209.0 ± 32.1	164.3 ± 22.6
ADFI /g	267.4 ± 49.7	299.2 ± 50.5	341.9 ± 95.2	315.2 ± 56.6
F/G	1.72 ± 0.22	1.71 ± 0.29	1.69 ± 0.23	1.97 ± 0.39
DR	0.17	0.17	0.18	0.09
**Day 11–15**				
ADG/g	389.6 ± 88.1	376.8 ± 99.6	383.0 ± 89.9	391.2 ± 50.9
ADFI/g	349.5 ± 113.8	321.0 ± 105.7	431.8 ± 95.0	317.0 ± 132.5
F/G	1.11 ± 0.12	1.17 ± 0.09	1.14 ± 0.22	1.26 ± 0.17
DR	0.125^c^	0.750^b^	0.063^a^	0.125^c^

a, b values within a row with different letters differ (*P* < 0.05).

c From day 1 to day 10 of the trial, piglets were fed the basal diet with or without α-terpineol or *B. coagulans* (BC). All diets were isocaloric. On day 11 to day 12, each piglet was orally inoculated with 1 × 10^10^ CFU of *E. coli* LMG194-STa. On the 15th day of the trial, all piglets were then slaughtered under anesthesia. Health indicators, such as body weight, food intakes, diarrhea incidence, were recorded during the entire experiment.

### 3.2. Plasma immunity, intestinal injury, and antioxidant ability parameters

Inflammatory, intestinal injury and redox indexes in plasma are present in Table 4. Compared with control group, ETEC infection significantly increased TNF-α and MDA concentration, decreased GSH-Px activity in plasma (*P* < 0.05). IL-1β and I-FABP concentration had a tend to be increased by ETEC infection. Compared with STa group, TPN+STa group significantly decreased the concentration of TNF-α, IL-1β, and MDA, and increased GSH-Px activity, I-FABP concentration had a tend to be decreased by α-TPN supplementation; BC+STa group significantly decreased the concentration of IL-1β, I-FABP and MDA in plasma (*P* < 0.05), and tend to increase GSH-Px activity.

**Table 4:** Inflammatory, intestinal injury, and redox index in plasma.

**Item**	**Control**	**STa**	**TPN+STa**	**BC+STa**
TNF-α (pg/mL)	35.05 ± 20.30^a^	72.45 ± 21.82^b^	46.11 ± 4.75^a^	69.75 ± 24.09^b^
IL-1β (pg/mL)	57.37 ± 12.15^ab^	77.69 ± 29.29^b^	43.70 ± 21.15^a^	46.31 ± 15.74^a^
I-FABP (pg/mL)	426.6 ± 89.76^ab^	494.0 ± 54.88^b^	469.4 ± 95.4^ab^	372.3 ± 66.96^a^
GSH-Px (U/mL)	512.9 ± 42.43^b^	427.3 ± 25.74^a^	551.56 ± 78.78^b^	485.9 ± 85.87^ab^
SOD (U/mL)	58.42 ± 4.27	57.58 ± 2.84	58.25 ± 5.73	55.73 ± 2.92
MDA (nmol/mL)	4.28 ± 1.17^a^	9.66 ± 1.69^c^	7.79 ± 2.06^b^	6.18 ± 1.97^b^

a, b, c values within a row with different letters differ (*P* < 0.05).

### 3.3. Intestinal morphology

**Figure 1:**
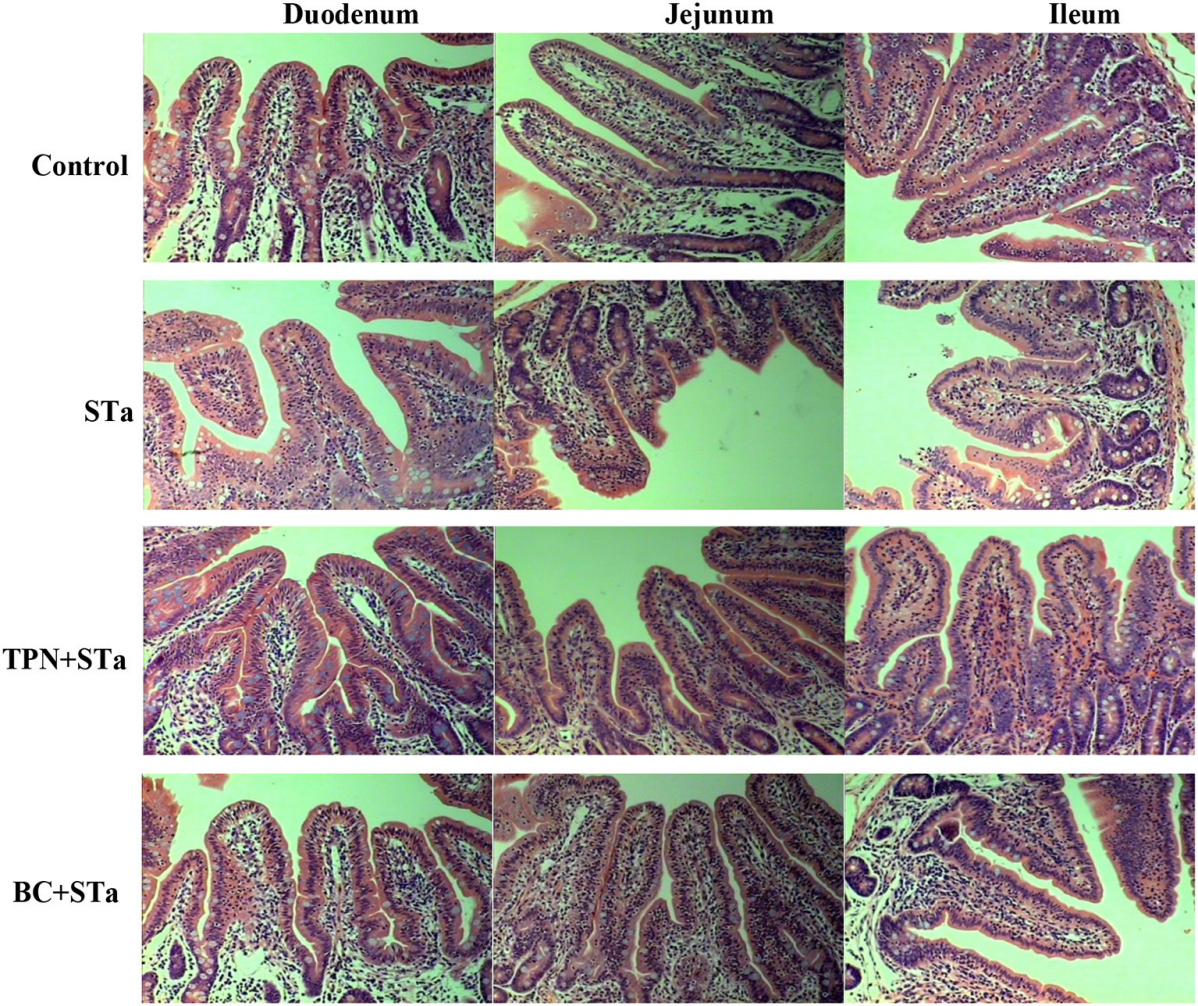
Intestinal mucosal morphology observed by H&E staining under an optical microscope. Magnification x 200.

The microscopic pictures of intestinal mucosal are shown in Figure 1, and the results of morphology is summarized in Table 5. The statistical analysis showed that compared to control group, ETEC infection significantly decreased villus height, width, surface area and the ratio of villus height to crypt depth in the duodenum, jejunum and ileum, increased crypt depth in the ileum (*P* < 0.05). Compared to STa group, α-TPN supplementation significantly increased villus surface area in the duodenum, increased villus height, surface area and villus height/crypt depth ratio in the ileum; *B. coagulans* supplementation significantly increased villus height in the duodenum, villus height, villus width and surface area in the jejunum, villus height and width and villus height/crypt depth ratio in the ileum, decreased crypt depth in the ileum (*P* < 0.05).

**Table 5:** Intestinal mucosal morphology.

**Item**	**Control**	**STa**	**TPN+STa**	**BC+STa**
**Duodenum**				
Villus height/μm	212.4 ± 20.07^b^	161.6 ± 25.07^b^	178.92 ± 23.47^b^	218.3 ± 31.47^b^
Crypt depth/μm	66.74 ± 13.93^b^	69.21 ± 14.26^b^	92.87 ± 17.58^b^	78.87 ± 3.53^b^
Villus height/crypt depth	3.60 ± 0.65^b^	2.52 ± 0.25^b^	2.03 ± 0.25^b^	2.79 ± 0.27^b^
Villus width/μm	185.3 ± 16.27^b^	150.2 ± 20.20^b^	163.5 ± 3.13^b^	156.2 ± 9.54^b^
Villus surface area/μm^b^	44951 ± 5101^b^	32403 ± 8757^b^	40342 ± 6189^b^	40879 ± 8800^b^
**Jejunum**				
Villus height/μm	285.5 ± 50.73^b^	255.4 ± 45.30^b^	276.3 ± 29.25^b^	310.0 ± 31.46^b^
Crypt depth/μm	76.52 ± 15.91^b^	65.25 ± 7.15^b^	67.18 ± 13.64^b^	89.22 ± 15.86^b^
Villus height/crypt depth	4.12 ± 0.49^b^	4.20 ± 0.73^b^	4.61 ± 0.69^b^	3.87 ± 0.58^b^
Villus width/μm	123.4 ± 15.65^b^	86.37 ± 10.94^b^	96.34 ± 15.65^b^	111.6 ± 18.77^b^
Villus surface area/μm^b^	35859 ± 8248^b^	27636 ± 5628^b^	32257 ± 6056^b^	42188 ± 9492^b^
**Ileum**				
Villus height/μm	188.7 ± 18.79^b^	159.7 ± 13.37^b^	183.3 ± 14.69^b^	174.8 ± 18.54^b^
Crypt depth/μm	82.80 ± 7.86^b^	95.71 ± 10.37^b^	92.37 ± 16.26^b^	81.50 ± 9.27^b^
Villus height/crypt depth	2.30 ± 0.33^b^	1.69 ± 0.27^b^	2.55 ± 0.72^b^	2.82 ± 0.34^b^
Villus width/μm	132.5 ± 6.20^b^	108.8 ± 5.99^b^	129.0 ± 6.06^b^	124.4 ± 11.79^b^
Villus surface area/μm^b^	32,651 ± 1,924	27,962 ± 2,714	29,563 ± 6,030	30,933 ± 6,805

a, b, c values within a row with different letters differ (*P* < 0.05).

### 3.4. Gene expression by real-time PCR

Expression level of genes associated with intestinal barrier, transport and metabolism are shown in Table 6. Compared to control group, ETEC infection significantly decreased expression level of genes matrix metalloproteinase-3 (*MMP3*) and Lipoprotein lipase (*LPL*) in the jejunum and colon, increased expression level of *villin, I-FABP, b*^0,+^*AT*, aquaporin *10* (*AQP10*), and insulin receptor (*INSR*) in the jejunum and colon as well as *KCNJ13* and *phosphoenolpyruvate carboxykinase 1 (PCK1)* in the jejunum (*P* < 0.05). Compared to STa group, α-TPN supplementation significantly increased expression level of *LPL* in the jejunum, decreased expression level of *villin, I-FABP*, b^0,+^ amino acid transporter (*b*^0,+^*AT*), *INSR*, and *PCK1* in the jejunum and colon as well as *MMP3* in the colon; *B. coagulans* supplementation increased expression level of *b*^0,+^*AT* in the colon, decreased expression levels of *villin, MMP3, AQP10, INSR* and *PCK1* in the jejunum colon and *I-FABP* in the colon (*P* < 0.05).

**Table 6:** Gene expression profiles in jejunum and colon.

**Item**	**Control**	**STa**	**TPN+STa**	**BC+STa**
**Jejunum**				
Villin	1.00 ± 0.18^b^	1.41 ± 0.34^a^	0.88 ± 0.20^bc^	0.82 ± 0.19^bc^
MMP3	1.00 ± 0.18^a^	0.76 ± 0.17^b^	0.76 ± 0.16^b^	0.48 ± 0.11^c^
I-FABP	1.00 ± 0.22^b^	1.36 ± 0.31^a^	0.99 ± 0.18^b^	1.45 ± 0.24^a^
b^0,+^AT	1.00 ± 0.25^b^	1.25 ± 0.21^a^	1.03 ± 0.22^b^	0.70 ± 0.18^c^
AQP10	1.00 ± 0.20^c^	4.45 ± 1.05^a^	3.87 ± 0.90^ab^	3.12 ± 0.79^b^
KCNJ13	1.00 ± 0.25^b^	1.71 ± 0.43^a^	1.44 ± 0.35^a^	1.56 ± 0.40^a^
INSR	1.00 ± 0.22^c^	2.44 ± 0.54^a^	1.56 ± 0.17^b^	1.20 ± 0.29^c^
LPL	1.00 ± 0.24^a^	0.48 ± 0.07^c^	0.78 ± 0.18^b^	0.49 ± 0.11^c^
PCK1	1.00 ± 0.25^c^	3.07 ± 0.52^a^	1.54 ± 0.36^b^	1.50 ± 0.38^b^
**Colon**				
Villin	1.00 ± 0.27^b^	1.48 ± 0.36^a^	0.94 ± 0.25^b^	0.98 ± 0.27^b^
MMP3	1.00 ± 0.20^a^	0.52 ± 0.11^b^	0.34 ± 0.08^c^	0.47 ± 0.10^b^
I-FABP	1.00 ± 0.22^c^	2.63 ± 0.73^a^	1.34 ± 0.35^bc^	1.14 ± 0.19^c^
b^0,+^AT	1.00 ± 0.23^d^	2.59 ± 0.57^b^	1.52 ± 0.29^cd^	3.52 ± 0.92^a^
AQP10	1.00 ± 0.22^b^	1.71 ± 0.43^a^	1.15 ± 0.28^b^	1.20 ± 0.25^b^
KCNJ13	1.00 ± 0.13^b^	1.11 ± 0.15^ab^	1.15 ± 0.25^ab^	1.22 ± 0.22^a^
INSR	1.00 ± 0.27^c^	1.41 ± 0.31^a^	0.93 ± 0.19^c^	1.10 ± 0.25^bc^
LPL	1.00 ± 0.20^a^	0.62 ± 0.14^b^	0.76 ± 0.16^b^	0.61 ± 0.15^bc^
PCK1	1.00 ± 0.16^a^	1.05 ± 0.24^a^	0.38 ± 0.09^c^	0.51 ± 0.12^c^

a, b, c, d values within a row with different letters differ (*P* < 0.05).

### 3.5. Protein expression by Western blot

**Figure 2:**
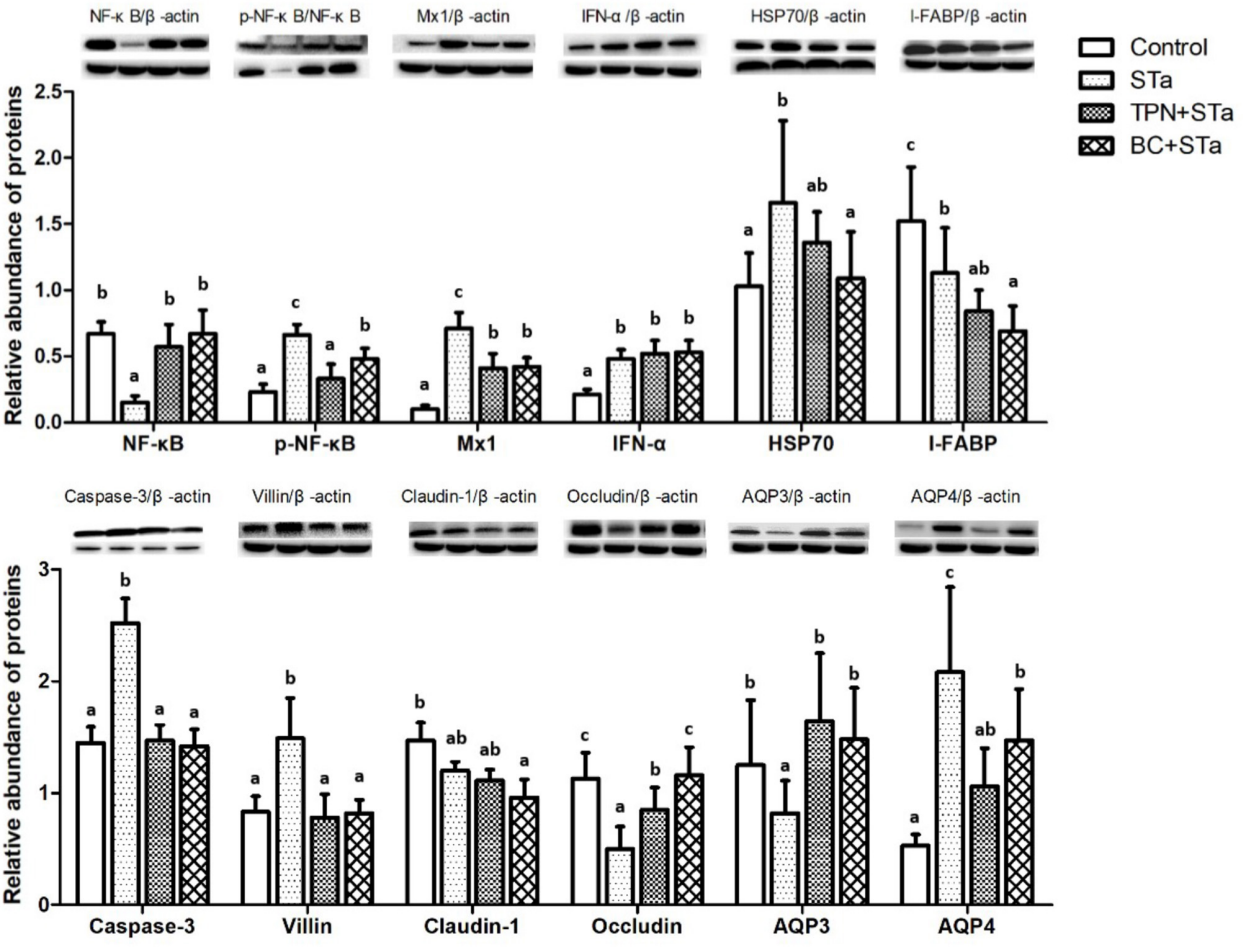
Effects of TPN and *B. coagulans* supplementation on the expression of proteins associated with intestinal barrier, immunity and metabolism in the jejunum of ETEC infected weaned piglets. Bars not sharing a common lowercase letter differ significantly (*p* < 0.05).

Expression level of proteins associated with intestinal barrier, immunity and metabolism in the jejunum are shown in Figure 2. Compared to control group, ETEC infection significantly increased expression levels of proteins p-NF-κB, Mx1, IFN-α, HSP70, Caspase-3, Villin, and AQP4, decreased expression level of NF-κB, I-FABP, Occludin and AQP3 (*P* < 0.05). Compared to STa group, α-TPN supplementation significantly increased expression level of proteins NF-κB, Occludin and AQP3, decreased expression level of p-NF-κB, Mx1, Caspase-3, Villin, and AQP4; *B. coagulans* supplementation increased expression level of NF-κB, Occludin and AQP3, decreased expression level of p-NF-κB, Mx1, HSP70, I-FABP, Caspase-3, Villin and AQP4 (*P* < 0.05).

## 4. Discussion

Diarrhea in piglets is one of the most challenging problems in the pig industry, which causes a huge economic loss nowadays (18). A major finding of this study is that diarrhea rate was markedly decreased by both α-TPN and *B. coagulans* supplementation, indicating that α-TPN and *B. coagulans* supplementation could effectively alleviate diarrhea in the weaned piglets. Furthermore, diarrhea rate in the TPN+STa group was lower than that in the BC+STa groups, suggesting that supplementation of α-TPN had a greater impact on alleviating diarrhea in the piglets infected with ETEC. The anti-diarrheal activity of α-TPN was also observed in mice, and this activity may due to its ability to block PGE2 and GM1 receptors (11). Further studies are needed to verify its anticholinergic action in piglets.

Intestinal morphology indexes such as villus height, surface area, crypt depth, and the ratio of villus height to crypt depth are the common indicators of intestinal morphologic development and intestinal morphological integrity (16). Usually, the increases in villus height, villus surface area, as well as the ratio of villus height to crypt depth reflect the improvement of intestinal absorption capacity and intestinal health (19). In this study, STa group decreased the height, width and surface area of villus and the ratio of villus height to crypt depth in the duodenum, jejunum and ileum, increased the crypt depth in the ileum, reflecting that ETEC infection markedly damaged intestinal integrity. However, both TPN+STa and BC+STa group increased villus height, width and surface area as well as the ratio of villus height to crypt depth, indicating that supplementation of α-TPN and *B. coagulans* can repair the intestinal injury caused by ETEC infection and enhance intestinal integrity. This was further supported by the finding that lower plasma I-FABP concentration was detected in BC+STa and TPN+ STa groups compared to STa group. As I-FABP leakages into the circulation from enterocytes only when intestinal mucosal damage occurs (20). Moreover, the improvement of intestinal morphology would partially explain the alleviation of diarrhea by α-TPN and *B. coagulans*.

Activation of the host immune system in response to pathogen infection results in the production of many cytokines that act as mediators of disease activity (21). In particular, proinflammatory cytokines, such as IL-1β, and TNF-α appear to play important roles in the pathophysiology of inflammatory diseases (22). In this study, STa group increased plasma TNF-α and IL-1β concentration, indicating that ETEC infection had obviously proinflammatory effect on piglets. Additionally, compared to STa group, TPN+STa group decreased the concentration of TNF-α and IL-1β; BC+STa group significantly decreased the concentration of IL-1β. These results suggested that both α-TPN and *B. coagulans* supplementation could relieve inflammation induced by ETEC infection and had anti-inflammatory properties.

Oxidant stress reflects the unbalance between the systematic phenomenon of reactive oxygen species and the capacity of biosystem to readily detoxicate the reactive intermediaries or to renovate the resulting injury (23). Nevertheless, cells protect themselves from hydroxyl radicals and other oxygenates by antioxidant enzymes, such as SOD and GSH-Px (24). MDA can induce noxious stress in cells and constitute homopolar protein adducts known as advanced lipoxidation end-products (ALEs), which is usually utilized as a marker to evaluate the oxidant stress levels (25). In the present study, STa group significantly increased MDA content and decreased GSH-Px activity in plasma, suggesting that ETEC infection could reduce the anti-oxidative capacity of piglets and induce oxidative stress. However, compared with STa group, both the TPN+STa and BC+STa group decreased the content of MDA and increased GSH-Px activity in plasma. These results indicated that α-TPN and *B. coagulans* supplementation could alleviant oxidative stress caused by ETEC infection, and supplementation of α-TPN and *B. coagulans* could enhance anti-oxidative capacity.

The injury and slow growth as well as the barrier function reducing of intestinal mucosa were probably associated with some genes such as villin, I-FABP, Occludin, and MMP3. Villin is one kind of actin binding protein and a marker of villus cell differentiation, which conduce to prop up the microfilaments of the microvilli of the mucosal villus (26). Occludin integrates such diverse processes as gene transcription, tumor suppression, and cell proliferation to modulate the intestinal mucosal structure and function (27). MMP3 as well as their inhibitors play a crucial role in the maintenance of extracorpuscular matrix homeostasis, which is expressed at high levels in the intestine of clinical IBD and celiac diseases (28). In response to stress, HSP70 is expressed at elevated levels to promote refolding and prevent aggregation of partially denatured proteins, thereby, protecting cells from injury (29). Results of this study showed that STa group decreased expression level of gene *MMP3* and protein Occludin, increased expression levels of gene *villin* and *I-FABP* and protein HSP70 and I-FABP. Whereas, compared with STa group, the TPN+STa and BC+STa group increased expression level of protein Occludin, which is beneficial for the repairment of intestinal barrier. The BC+STa group decreased expression level of protein HSP70, indicating that *B. coagulans* supplementation could promote the recovery from ETEC infection.

Caspase-3 is one of the key components of the apoptotic pathway in the small intestine, this protein is either partially or totally responsible for the proteolytic cleavage of many key proteins (30). The expression of caspase-3 was increased by ETEC infection whereas decreased by α-TPN and *B. coagulans* supplementation, which might contribute to enterocyte proliferation.

NF-κB is involved in activation of an exceptionally large number of genes in response to infections, inflammation, and other stressful situations requiring rapid reprogramming of gene expression (31). NF-κB is normally sequestered in the cytoplasm of non-stimulated cells and consequently must be translocated into the nucleus to stimulate gene transcription. The subcellular location of NF-κB is controlled by a family of inhibitory proteins, IκBs, which bind NF-κB and mask its nuclear localization signal, thereby preventing nuclear uptake (32). Exposure of cells to a variety of extracellular stimuli leads to the rapid phosphorylation, ubiquitination, and ultimately proteolytic degradation of IκB, which frees NF-κB to translocate to the nucleus where it regulates gene transcription (33). Mx proteins form a family of interferon (IFN)-induced GTPases with potent antiviral activity against various single-stranded RNA viruses in mammals (34). In the present study, STa group decreased expression level of NF-κB, increased expression levels of Mx1 and p-NF-κB, whereas both the TPN+STa and BC+STa group increased expression level of NF-κB, decreased expression level of Mx1 and p-NF-κB. The decline of p-NF-κB expression was in accordance with the decrease of TNF-α and IL-1β concentration in plasma. Together, these results demonstrated that ETEC infection activated the immune response in the piglets, while α-TPN and *B. coagulans* supplementation could alleviate the immune stress in the piglets during ETEC infection.

AQP4 and AQP10 are two of the most important water channel proteins and mainly distributed in intestinal epithelial cells and facilitated the transport of water across epithelial cell (35). KCNJ13 is an ATP-dependent calcium channel that transports calcium out of cells, which plays a very considerable role in calcium homeostasis (36). b^0,+^AT plays a role in the high-affinity and sodium-independent transport of cystine and neutral and dibasic amino acids and appears to function in the reabsorption of cystine in the kidney tubule (37). Increased level of genes *b*^0,+^*AT, AQP10, KCNJ13*, and protein AQP4 were observed in the STa group, indicated that ETEC infection could increase intestinal transportation, which could be ascribed to the damage of intestinal barrier and may the mechanism of diarrhea. Additionally, α-TPN and *B. coagulans* supplementation alleviated the intestinal transport dysfunction in the piglets during ETEC infection, as presented by decreased level of gene *b*^0,+^*AT* in the TPN+STa group and gene *AQP10* in the BC+STa group, as well as protein AQP4 in both groups.

INSR is a transmembrane receptor which can binds insulin, IGF-I, and IGF-II and belongs to the large class of tyrosine kinase receptors. Binding of insulin or other ligands to this receptor activates the insulin signaling pathway, which regulates glucose uptake and release, as well as the synthesis and storage of carbohydrates, lipids and protein (38). PCK1 is a main control point for the regulation of gluconeogenesis and can be regulated by insulin, glucocorticoids, glucagon, cAMP, and diet (39). LPL is expressed in heart, muscle, and adipose tissue, which functions as both triglyceride hydrolase and ligand/bridging factor of receptor-mediated lipoprotein uptake (40). This study observed that STa group decreased expression level of *LPL*, increased expression levels of *INSR* and *PCK1*; whereas both the TPN+STa and BC+STa group decreased the level of *INSR* and *PCK1* and the TPN+STa group increased expression level of *LPL*. The opposite results on metabolic regulation between ETEC and two supplements indicated the beneficial impact of α-TPN and *B. coagulans* on intestinal metabolism in piglets during ETEC infection.

## 5. Conclusion

The present study investigated the beneficial impact of dietary supplementation of α-TPN and *B. coagulans* in piglets infected with ETEC. Both α-TPN and *B. coagulans* supplementation could relieve diarrhea, inflammation, intestinal injury, oxidative and immune stress caused by ETEC infection, by means of modulation of genes and proteins associated with intestinal barrier, immune response, transport and metabolism. Findings of this study demonstrated that both α-TPN and *B. coagulans* can be used against ETEC infection, and could be considered as ideal alternatives to antibiotics.

## Data availability statement

The original contributions presented in the study are included in the article/supplementary material, further inquiries can be directed to the corresponding author.

## Ethics statement

The animal study was reviewed and approved by Animal Care and Use Committee at Wuhan Polytechnic University.

## Author contributions

YH contributed to conception and design of the study. TW and QZ conducted the experiments and wrote the draft of the manuscript. HX and PL assisted the animal experiment. DZ and LW performed the statistical analysis. YH and DY revised the manuscript. All authors read and approved the submitted version.
